# Glass-forming property of hydroxyectoine is the cause of its superior function as a desiccation protectant

**DOI:** 10.3389/fmicb.2014.00150

**Published:** 2014-04-04

**Authors:** Christoph Tanne, Elena A. Golovina, Folkert A. Hoekstra, Andrea Meffert, Erwin A. Galinski

**Affiliations:** ^1^Institute of Microbiology and Biotechnology, Rheinische Friedrich-Wilhelms-University BonnBonn, Germany; ^2^Laboratory of Plant Physiology, Wageningen UniversityWageningen, Netherlands

**Keywords:** hydroxyectoine, desiccation, glass transition temperature, enzyme stabilization, ESR, FTIR

## Abstract

We were able to demonstrate that hydroxyectoine, in contrast to ectoine, is a good glass-forming compound. Fourier transform infrared and spin label electron spin resonance studies of dry ectoine and hydroxyectoine have shown that the superior glass-forming properties of hydroxyectoine result from stronger intermolecular H-bonds with the OH group of hydroxyectoine. Spin probe experiments have also shown that better molecular immobilization in dry hydroxyectoine provides better redox stability of the molecules embedded in this dry matrix. With a glass transition temperature of 87°C (vs. 47°C for ectoine) hydroxyectoine displays remarkable desiccation protection properties, on a par with sucrose and trehalose. This explains its accumulation in response to increased salinity and elevated temperature by halophiles such as *Halomonas elongata* and its successful application in ``anhydrobiotic engineering'' of both enzymes and whole cells.

## INTRODUCTION

**FIGURE 1:**
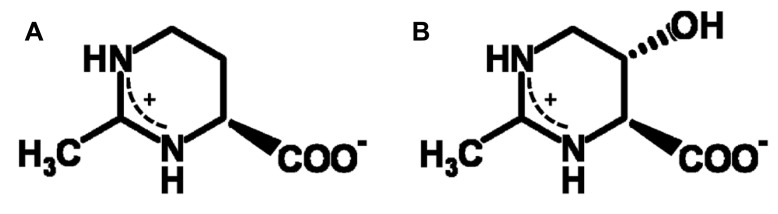
**Chemical structure of ectoine (A) and hydroxyectoine (B)**.

Compatible solutes (organic osmolytes) are low-molecular mass water-binding organic solutes, which are accumulated in the cytoplasm of halophiles for osmotic equilibrium, either as a replacement for or in combination with inorganic salts. They are also known as versatile stress-protecting compounds, in particular for the stabilization of proteins, membranes and whole cells. The cyclic amino acid derivative ectoine (**Figure 1A**) is one of the most common compatible solutes among halophilic heterotrophic *Bacteria* and has found diverse applications, above all as ingredient of skin care products (Graf et al., 2008). Organisms able to synthesize ectoine are often also able to convert this compound into *S,S*-1,4,5,6-tetrahydro-2-methyl-5-hydroxy-pyrimidine-4-carboxylate, hydroxyectoine (**Figure 1B**), by means of a 2-oxoglutarate dependent non-heme-iron(II) containing dioxygenase (Bursy et al., 2007; Reuter et al., 2010; Widderich et al., 2014). In *Chromohalobacter salexigens*, a member of the *Halomonadaceae*, the relative proportion of the hydroxylated derivative increases with salinity and/or temperature (Garcia-Estepa et al., 2006; Vargas et al., 2008). It has long been known that hydroxyectoine is a superior stress protectant against desiccation for both whole cells and enzymes (Lippert and Galinski, 1992; Louis et al., 1994). This knowledge has subsequently been applied in “anhydrobiotic engineering” of *Escherichia coli* and *Pseudomonas putida* (Manzanera et al., 2002, 2004a,b). In addition, a comparative enzyme protection study with heat-stabilizing compounds from extreme thermophiles (Borges et al., 2002) has revealed superb heat-stabilizing properties. However, the biophysical basis of the difference between ectoine and hydroxyectoine, of which only the latter is a good heat and desiccation protectant, has until now not been resolved. The sugars sucrose and trehalose, on the other hand, are well known desiccation protectants in all domains of life and their remarkable function has been linked to the ability to form glasses, which in selected cases ensures conservation of biological functions in an (almost) completely dry state. This phenomenon of “anhydrobiosis” (life without water; Clegg, 2001) is apparent in many higher forms of life (e.g., seeds, resurrection plants, tardigrada, the chironomid *Polypedilum vanderplanki*). Cytoplasmic glasses are considered one of the main mechanisms of desiccation tolerance (Crowe et al., 1998; Hoekstra et al., 2001; Potts, 2001; Buitink and Leprince, 2004; Ballesteros and Walters, 2011). The outstanding role of disaccharides (possibly in combination with intrinsically disordered proteins, IDPs) has given rise to a number of biophysical models, of which the “water entrapment” (Belton and Gil, 1994; Cottone et al., 2002) and “anchorage” hypothesis (Allison et al., 1999; Francia et al., 2008) are the most comprehensive because they encompass glass-formation of solutes and simultaneous entrapment of small water clusters, possibly anchored to critical sites at the interface with biomolecules.

The preservation of biological structures and bioactivity over a long period of time is a main challenge in life science with impacts on medicine, pharmacy and biohybrid technologies (e.g., biosensors). As the halophilic *Halomonas elongata* is unable to synthesize trehalose and/or sucrose, but appears to convert ectoine into hydroxyectoine in response to heat and water stress, we investigated whether this provides an alternative adaptation strategy for survival of cells and biomolecules in the dry state. This required a biophysical comparison of the glass-forming abilities of ectoine and hydroxyectoine and, in particular, the thermal stability of anhydrous glasses as characterized by the glass transition temperature (Tg). As intermolecular H-bonding plays an essential role in thermal properties of glasses (Omayu et al., 2008; Zhou et al., 2013), temperature-controlled infrared spectroscopy is widely used to study hydrogen bonds in solids. A compatible approach in the study of anhydrous glasses is spin probe electron spin resonance (ESR). As the motion of spin probe molecules is influenced by their intermolecular H-bonding with surrounding molecules, the dynamics of spin probe molecules is obtained from the analysis of the shape of their ESR spectra. Both approaches were used here to characterize differences in glass properties of ectoine and hydroxyectoine.

High molecular immobilization in glasses provides chemical stability of the system due to restricted molecular diffusion. Chemical stability together with structural stability are the main factors which determine survival of organisms in the dry state. The spin probe approach provides an opportunity to obtain information about molecular immobilization and redox activity from the same ESR spectra. Spin probe molecules can participate in different redox-reactions resulting in non-paramagnetic species (Belkin et al., 1987). Therefore, the spin probe approach is a unique technique which allows direct observation of the relationships between structural and chemical stability of dry systems.

## MATERIALS AND METHODS

### CHEMICALS

Antibiotic broth medium No. 3 (complex medium) was purchased from Oxoid LTD (Hampshire, Great Britain). Pyruvic acid, sucrose, KH_2_PO_4_, KOH, and (NH_4_)_2_SO_4_ were purchased from Roth (Karlsruhe, Germany). D-glucose, MgSO_4_·7 H_2_O, and FeSO_4_·7 H_2_O were obtained from Merck (Darmstadt, Germany). Trehalose and NaCl were purchased from Fluka (Buchs, Schwitzerland). Ectoine (≥99%) for lactate deydrogenase stress experiments was purchased from bitop AG (Witten, Germany). Hydroxyectoine (≥99%) for lactate dehydrogenase (LDH) stress experiments was isolated from *H. elongata* strain DSM 2581^T^ in our laboratories. Freeze-dried LDH (rabbit muscle) and nicotinamide adenine dinucleotide were purchased from Sigma (Steinheim, Germany). Phosphate buffered saline (PBS) was purchased from AppliChem (Darmstadt, Germany). Ectoine (≥99.0%) and hydroxyectoine (≥98%) for spin lable and Fourier transform infrared (FTIR) experiments were purchased from Sigma (USA). Perdeuterated spin probe Tempone-d_16_ (4-Oxo-2,2,6,6-tetramethylpiperidine-d_16_-1-oxyl; **Figure 7** inset) was a kind gift of Prof. I. Grigoriev (Institute of Organic Chemistry of the Russian Academy of Sciences, Novosibirsk, Russia).

### STRAINS, MEDIA, AND CULTIVATION

*Halomonas elongata* strain DSM 2581^T^ was obtained from DSMZ (Braunschweig, Germany). Complex media were used for precultures (5.0 g/L peptone, 1.5 g/L yeast extract, 1.5 g/L “Lab-Lemco” powder, 1.0 g/L glucose, 3.68 g/L K_2_HPO_4_, 1.32 g/L KH_2_PO_4_ and 150 g/L NaCl; pH was adjusted to 7.4). Minimal media MM63 were used for second pre- and for main culture [13.61 g/L KH_2_PO_4_, 4.21 g/L KOH, 0.25 g/L MgSO_4_·7 H_2_O, 1.98 g/L (NH_4_)_2_SO_4_, 0.0011 g/L FeSO_4_·7 H_2_O, 5 g/L glucose, and the required amount of NaCl, pH was adjusted to 7.4; Larsen et al., 1987].

Bacterial cultures were grown in shake flasks. Cell growth was tracked by optical density measurement of incubated media at a wavelength of 600 nm. For solute content analysis *H. elongata* was grown in MM63 with 10 or 15% NaCl (w/w) at 30, 40, and 45°C, respectively. In addition, to surpass its temperature limit of growth, a thermal shock from 37 to 50°C was applied in the mid exponential growth phase. For the determination of desiccation survival, *H. elongata* was grown in two main cultures MM63 with 15% NaCl at 30°C. In the mid exponential growth phase one of the main cultures was shocked to 50°C the other remained at 30°C. After 4 h in stationary phase samples were taken for survival experiments.

### SOLUTE CONTENT ANALYSIS

Bacterial cell material was harvested in the stationary growth phase and dried in a rotational vacuum concentrator (SpeedVac) for at least 8 h at 45°C and 10 mbar. Solute extraction was achieved following the Bligh and Dyer protocol (Bligh and Dyer, 1959), as modified by Galinski and Herzog (1990). Homogenized samples (30 mg) of dried bacterial biomass were extracted by vigorous shaking (10 min) with 500 μL of modified Bligh and Dyer solution [methanol/chloroform/water 10:5:4 (v/v)]. Following the addition of 130 μL chloroform and 130 μL water, phase separation was assisted by centrifugation at approximately 9300 *g* (5 min) and the resulting polar upper phase was used for high performance liquid chromatography (HPLC) analysis. For the detection of neutral zwitter-ionic or polar uncharged water soluble compatible solutes an aminopropyl-modified silica column was used (Grom-Sil Amin-1PR, 3 μm, 125 × 4 mm, LiChrocart-System, Alltech Grom GmbH). The isocratic eluent was 80% acetonitrile-water (v/v) at a flow rate of 1 mL/min. A combination of UV- and RI-detector was used for peak identification and solute quantification.

### DESICCATION-SURVIVAL EXPERIMENT

Stationary phase samples were diluted with glucose-free medium to an optical density of 0.1. From this dilution, 100 μL were taken to determine the initial cell number (threefold). The same volume (100 μL) of each sample was transferred into both a closed 1.5 mL microcentrifuge tube (undried control) and an open 1.5 mL microcentrifuge tube. The samples were then subjected to vacuum drying for 3 h at 45°C and 10 mbar in a SpeedVac (RVC 2-25 CD plus, Christ, Osterode am Harz). Subsequently, dried samples (from open microcentrifuge tubes) were rehydrated by adding 1 mL of glucose-free MM63. Samples were further diluted to 10^-6^ or 10^-7^ for viable cell number determination. This procedure was repeated three times for every sample. Appropriate dilutions of the samples were plated on a complex medium agar plate with 15% NaCl. After 48 h of incubation at 30°C colony forming units (CFU) were counted and expressed as percentage of initial cell number.

### CASTING OF SOLUTE GLASS MATRICES

To cast solute glass matrices 3 μL of 2 M solute solution in ultrapure water were placed on a clean polystyrene plate and dried at 60°C for 2 h. Transmission light microscopy was used for optical characterization of glass matrices.

### LACTATE DEHYDROGENASE (LDH) STRESS EXPERIMENT AND ACTIVITY ASSAY

Ten microliter of 2 M solute solution or ultrapure water with 0.05 mg/mL LDH were placed carefully into separated wells of a 96 well plate. Solidification of solute solutions was accomplished by air drying at 60°C for 2, 4, and 6 h. Rehydration to the original volume was accomplished within 2 min of incubation in PBS buffer, pH 7.5 (AppliChem, Darmstadt) at room temperature. The working volume (200 μL) for the activity determination of undried (control) and rehydrated LDH solute matrices was obtained by diluting (20-fold) to a final enzyme concentration of 2.5 μg/mL LDH with 160 μL PBS buffer (pH 7.5), 20 μL 10 mM pyruvic acid (final concentration 1 mM) and by addition of 20 μL of 7.5 mM NADH/H^+^ (final concentration of 0.75 mM) to start the reaction. Decrease in absorbance at 340 nm was monitored.

### SPIN LABEL EXPERIMENTS

Tempone was added to aqueous solutions of ectoine or hydroxyectoine (32 mg/mL) in a final concentration of 1 mol%, so that in a dry state the proportion of (hydroxy) ectoine: Tempone = 100:1. At such proportion the concentration broadening of the ESR spectra would not be observed under conditions of uniform distribution of spin probe molecules. Samples of labeled solution (100 μL) were spread over chemically inert glass beads (80–110 μm diameter) on a glass slide and allowed to dry for 5 days a in a stream of dry air (3% RH) at room temperature in an air-dry box. The dried material was transferred to 2-mm capillaries in an air-dry box (3% RH), to prevent rehydration on air, and then flame-sealed.

The capillary with the sample was placed in an ESR quartz tube for spectrum recording. ESR spectra were recorded with an X-band ESR spectrometer (Elexsys model E 500; Bruker Analytik, Rheinstetten, Germany) equipped with a temperature unit using regular air within the temperature range 295–400K and liquid N_2_ for temperatures below 295 K. The spectra were recorded at 5° increments with equilibration for 1 min at each temperature. The scan range was 100 G for all spectra. To prevent over-modulation and saturation of ESR signal, the modulation amplitude was 2.5 G for solid-like spectra and 1 G for fluid-like spectra. The microwave power was limited to 5 mW.

### FTIR EXPERIMENTS

Small volumes (5 μL) of aqueous solution of ectoine or hydroxyectoine (32 mg/ml) were dried on circular CaF_2_ windows (2 × 13 mm) in a stream of dry air (3% RH) at room temperature in an air-dry box. Although most of the water was removed fast, the samples were further air-dried at 3% RH for 5 days in order to achieve equilibrium water potential. Each sample was hermetically sealed between two CaF_2_ windows using a rubber O-ring and mounted into a temperature-controlled brass cell.

Infrared spectra of dry ectoine and hydroxyectoine were obtained with a Perkin-Elmer series 1725 FTIR spectrometer equipped with an external beam facility to which a Perkin-Elmer IR-microscope was attached. The microscope was equipped with a narrow band mercury-cadmium-telluride liquid nitrogen-cooled IR-detector. The samples between two CaF_2_ windows were tightly mounted into a temperature-controlled brass cell that was cooled by liquid nitrogen. The temperature of the cell was regulated by a computer-controlled device that activated a liquid nitrogen pump in conjunction with a power supply for heating the cell. The temperature of the sample was recorded separately using two PT-100 elements that were located very close to the sample windows. The optical bench was purged with dry CO_2_-free air. Spectra were recorded starting with the lowest temperature with a scanning rate of 1.5°C/min. The acquisition parameters were: 4 cm^-1^ resolution, 32 co-added interferograms, 3500–1000 cm^-1^ wavenumber range.

Spectral analysis and display were carried out using the Infrared Data Manager Analytical software, version 3.5 (Perkin–Elmer). The temperature-induced changes in dry ectoine and hydroxyectoine matrixes were monitored by observing the position of the bands around 1388 and 1088 cm^-1^. The band around 1388 cm^-1^ is present in both ectoine and hydroxyectoine, while the band around 1088 cm^-1^ is present only in hydroxyectoine (**Figure 11B**). The band positions were calculated as the average of spectral positions (*n* = 50) at 75% of the total peak height (Wolkers et al., 1998). Breaks in the temperature dependence of this peak position were determined as a point of intersection of two regression lines below and above the temperature of the break (**Figure 12A**).

All ESR and FTIR experiments were conducted on the same samples prepared under the same conditions. Each model experiment was repeated at least twice, and the results of the single experiments are presented.

## RESULTS

### INFLUENCE OF WATER AND TEMPERATURE STRESS ON INTRACELLULAR ECTOINE LEVELS

**FIGURE 2:**
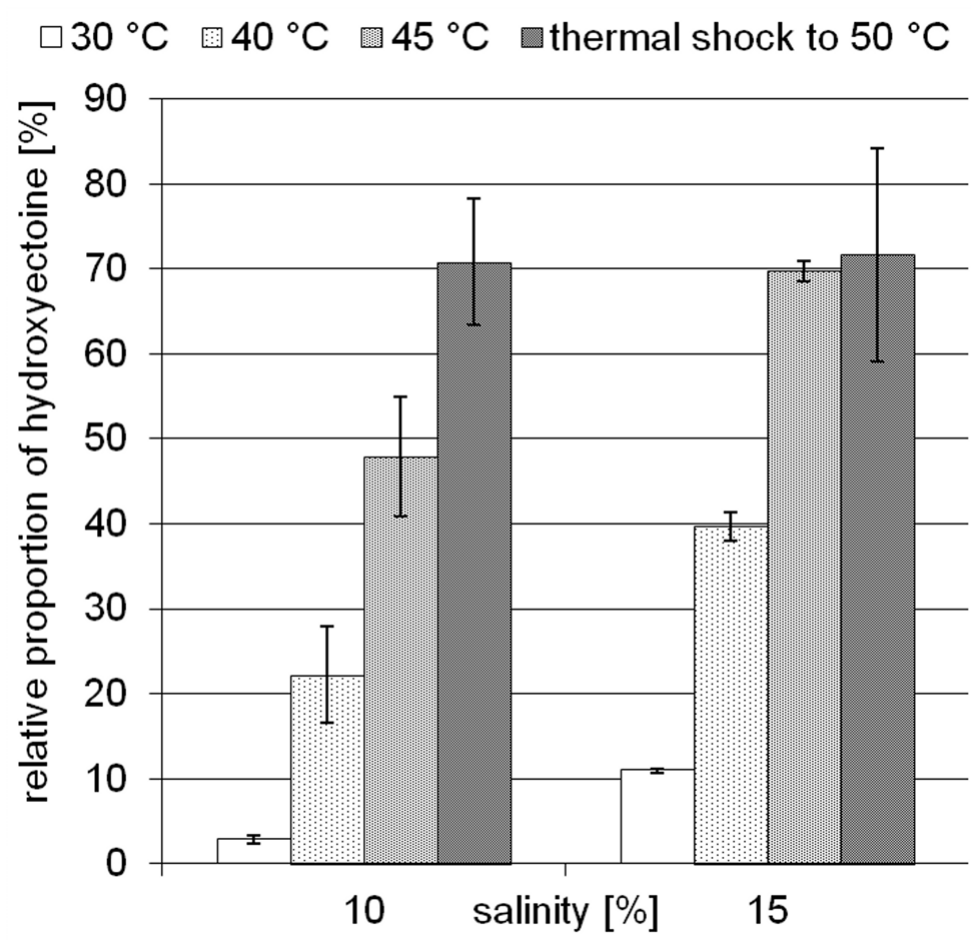
**Influence of temperature and salinity on the relative proportion of hydroxyectoine in *H.* elongata.** Error bars represents lowest and highest value of duplicate measurements for both salinities at 30°C and for 15% NaCl at 45°C; all others represent standard deviations of three replicates (in the case of 15% NaCl, upshock) or at least four replicates.

The moderately halophilic (optimum 3–5% NaCl) but extremely halotolerant *H. elongata* employs the compatible solutes ectoine/hydroxyectoine for osmotic adaptation by increasing their cytoplasmic concentration in a near-linear fashion. At a salinity of 15% NaCl, *H. elongata* experiences severe water stress and, as a consequence, its growth rate is reduced to 0.1 (approximately 25% of maximum; Dötsch et al., 2008). In contrast to other members of the family *Halomonadaceae* (e.g., *C. salexigens*), *H. elongata* is not able to synthesize the well-known desiccation protectant trehalose, which makes it a good model for investigating the role of hydroxyectoine. As depicted in **Figure 2**, increase in both temperature and salinity leads to a higher relative proportion of hydroxyectoine. At its maximum growth temperature of 45°C and a salinity of 15% NaCl, the hydroxyectoine level reached 70% of total ectoines. Although the organism is unable to grow at 50°C from inoculum, it was possible to increase the hydroxyectoine level even further by upshock experiments (i.e., raising the temperature in mid-exponential phase to 50°C). We used the combination of high salinity (15%) and temperature upshock to simulate a dehydration event and investigated its impact on survival rates of *H. elongata*.

### DRY STABILIZATION OF *H. elongata* CELLS AT ELEVATED HYDROXYECTOINE LEVELS

**FIGURE 3:**
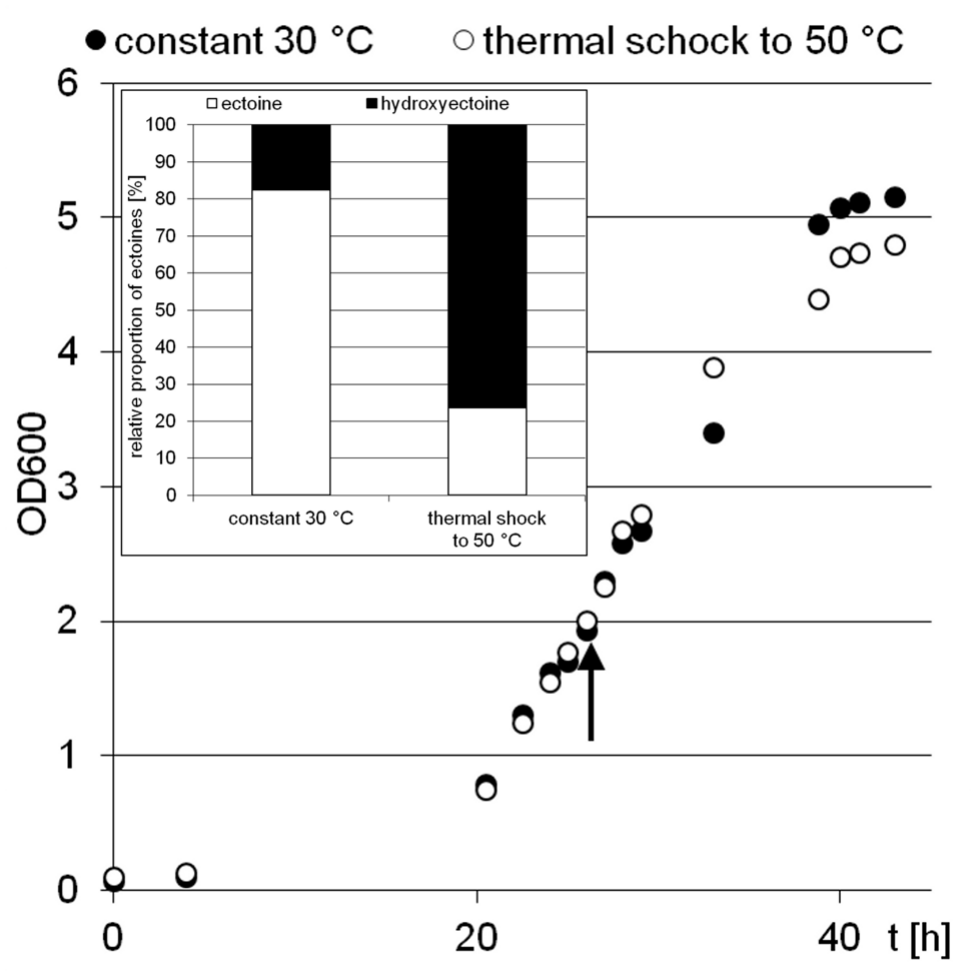
**Growth of *H. elongata* in minimal medium MM63 with 15% sodium chloride.** The experiment was performed with two parallel culture, one at constant 30°C (black dots), the other with a rapid temperature upshock at OD 2 (arrow) from 30 to 50°C (white dots). The inset shows the relative proportions of ectoines in bacterial cells at the early stationary growth phase (point of harvest).

**FIGURE 4:**
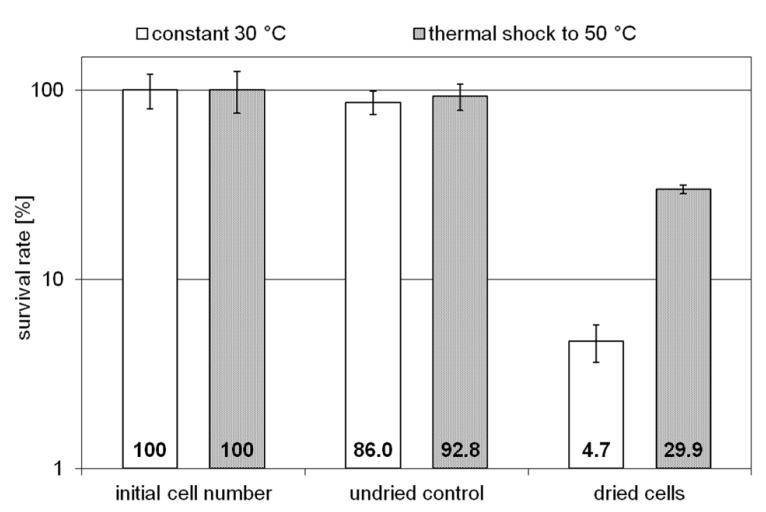
**Survival rates of unstressed *H. elongata* cells (initial cell number), undried control cells and cells which survived harsh drying for 3 h at 45 °C and 10 mbar (error bars show standard deviations of triplicates)**.

Two *H. elongata* cultures were grown at 15% NaCl and 30°C to an optical density of approximately 2, when one of them was heat shocked to 50°C (arrow in **Figure 3**). Subsequently, the solute content of both cultures was analyzed at early stationary phase. It is clearly seen that the applied temperature increase had little effect on the organisms growth and yield. The relative proportion of hydroxyectoine, however, increased from approximately 17% (at 30°C) to approximately 75% (at 50°C; **Figure 3**, inset), indicating that the conversion of ectoine into hydroxyectoine is enhanced further by temperature upshock. To compare the survival rates of desiccated stationary phase cells, colony-forming units of initial cell numbers (control before drying process), undried controls (from closed vials) and dried samples (vacuum drying for 3 h at 45°C and 10 mbar) were determined (**Figure 4**). It was demonstrated that heat-stressed cells with 75% hydroxyectoine had much higher survival rates. A survival rate of nearly 30% was achieved as compared to only 4.7%, for untreated cells. Although it cannot be excluded that other adaptational processes may also be responsible for improved survival of heat-shocked cells (see Discussion), we concluded that the hydroxylation of ectoine plays an important part in this improved desiccation survival. Provided that a simple hydroxylation step is indeed able to alter the properties of a common compatible solute (ectoine) in such a way as to provide desiccation protection, then drying of the model enzyme LDH should show a similar response and the protective effect of hydroxyectoine should compare favorably with the well-known desiccation protectants sucrose and trehalose.

### STRESS PROTECTION BY HYDROXYECTOINE DURING HEATING/DRYING OF LACTATE DEHYDROGENASE (LDH)

**FIGURE 5:**
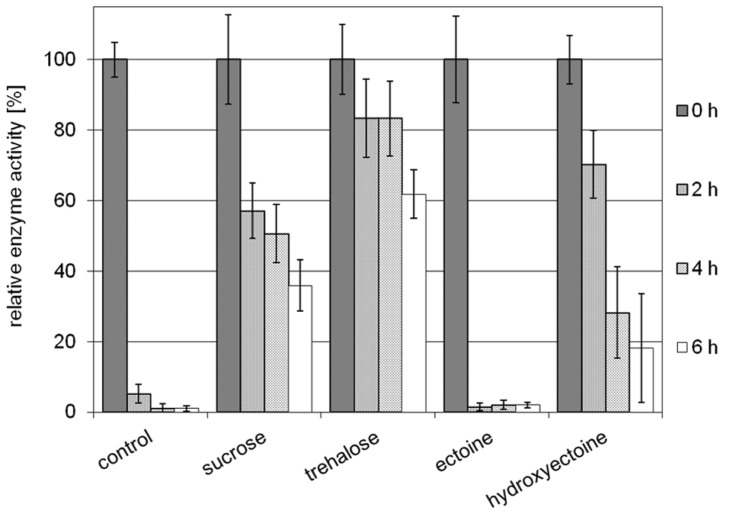
**Relative enzyme activities of LDH after prolonged air drying at 60°C for 2, 4, and 6 h.** Activities of unstabilized enzyme (control) and of enzyme protected by sucrose, trehalose, ectoine, and hydroxyectoine, respectively, are shown (error bars show standard deviations of at least four to a maximum of six replicates).

Stabilization of model enzyme LDH by compatible solutes against heat and freeze-drying has been investigated before, and for both stress factors the superiority of hydroxyectoine over ectoine has been demonstrated (Lippert and Galinski, 1992; Borges et al., 2002). Here a small volume (10 μL) of LDH at a concentration of 0.05 mg/mL in 2 M solutes (ectoine, hydroxyectoine, sucrose, and trehalose, respectively) was exposed to air-drying at 60°C. The dried protein was subsequently diluted in buffer and checked for residual activity. As shown in **Figure 5**, the unprotected enzyme loses approximately 95% of its original activity after 2 h of drying. Prolonged drying destroyed activity almost completely. It is worthy of note that the presence of ectoine had no stabilizing effect under the conditions employed, whereas hydroxyectoine after 2 h drying at 60°C displayed a residual activity of approximately 70%, which lies between the values of sucrose (58%) and trehalose (83%) as benchmarks. Upon further drying, however, the stabilizing effect of hydroxyectoine declined more rapidly than with disaccharides. Nonetheless the observed differences between ectoine (no stabilization) and hydroxyectoine (on a par with disaccharides after 2 h of drying) are remarkable and put hydroxyectoine into the same category as the glass-forming disaccharides. We therefore expanded our comparison of both ectoines to include their glass-forming abilities.

### GLASS-FORMING ABILITIES OF ECTOINES

**FIGURE 6:**
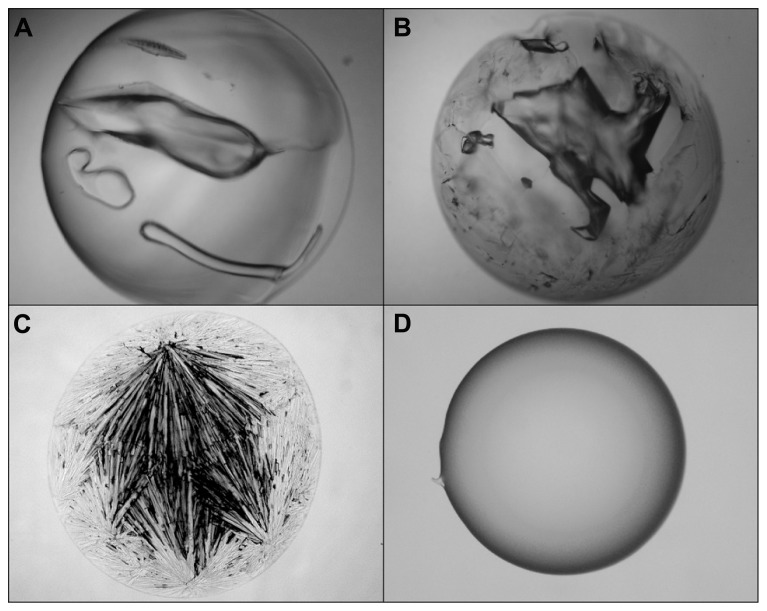
**Light microscopic photographs of solute matrices which were casted from 2 M solutions of trehalose (A), sucrose (B), ectoine (C), and hydroxyectoine (D) by air drying of 10 μL at 60°C for 2 h**.

A simple glass-casting experiment from 2 M solution at 60°C disclosed a striking difference between both ectoines (**Figures 6C,D**). While hydroxyectoine formed a clear and transparent solid, visual examination of ectoine samples revealed an inhomogeneous structure with crystalline inclusions, indicating a mixture of glassy and crystalline states. It can also be seen that the benchmark glass-formers sucrose and trehalose formed solids with cracks under the drying conditions employed (**Figures 6A,B**). These were never observed with solid hydroxyectoine samples. Thus it can be concluded that hydroxyectoine, in contrast to ectoine, is a good glass former and that this property is probably related to the superior desiccation protection of hydroxyectoine on biological structures.

### SPIN PROBE STUDY OF GLASS PROPERTIES OF DRY ECTOINE AND HYDROXYECTOINE

**FIGURE 7:**
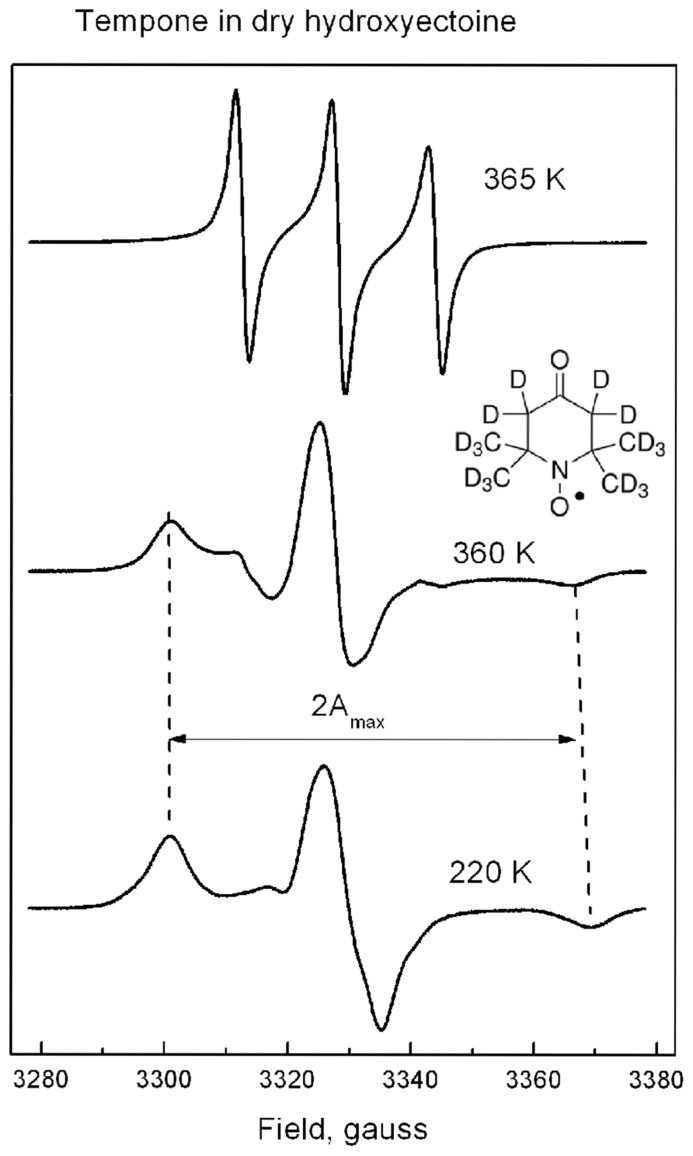
**The shape of ESR spectra of Tempone in dry hydroxyectoine at 220, 360, and 365 K.** Inset – the molecular structure of perdeuterated Tempone. The way of calculation of 2A_max_ is indicated.

Hydrogen bonds and packing density are the key factors which determine the properties of anhydrous glasses (Omayu et al., 2008; Wang et al., 2009; Zhou et al., 2013). Spin probe Tempone as a reporter molecule was used to study the glass properties of dry ectoine and hydroxyectoine. Tempone is a small water-soluble stable free radical, which has the shape of a sphere with a radius around 3 Å. It has one ketone group >C=O (**Figure 7**, inset), which can be an acceptor for H-bonds. The uncoupled electron nitroxide group N-O is surrounded by 4 bulky methyl groups (**Figure 7**, inset) and is probably less available for H-bonding. One molecule of ectoine can provide two donors for hydrogen bonds – two N-H groups from ring N – and one acceptor C=O from COO^-^ group (**Figure 1**). Hydroxyectoine has one additional potential donor. This is a hydroxyl group -OH, which is attached to the heterocyclic ring (**Figure 1**). The H-bonds between >C=O of Tempone and N-H groups of both ectoine and hydroxyectoine are less strong than the H-bond between >C=O of Tempone and OH group of hydroxyectoine because oxygen is more electronegative than nitrogen. Being hydrogen-bonded with ectoine and hydroxyectoine, the spin probe molecule moves collectively with the solute molecules as long as such H-bonds exist. Tempone has an ESR spectrum consisting of three lines due to hyperfine interactions of its uncoupled electron with nitrogen spin (Knowles et al., 1976). Because of spectral anisotropy, ESR spectra of spin probes are sensitive to motion and are, therefore, suitable for a temperature-dependent study of molecular immobilization in a dry matrix caused by H-bonds.

Tempone in dry hydroxyectoine has a solid-like spectrum up to 360 K (**Figure 7**). This spectral shape is typical for highly immobilized spin probe molecules. The distance between outer extremes 2A_max_ (**Figure 7**) is used to characterize the degree of immobilization of the nitroxide moiety of the spin probe (Knowles et al., 1976). Above 360 K a sudden change in the spectral shape is observed (**Figure 7**). The spectrum of Tempone at 365 K in **Figure 7** has three equidistant narrow lines. Such a spectral shape is typical for fast isotropic rotation of the spin probe molecule (Knowles et al., 1976).

**FIGURE 8:**
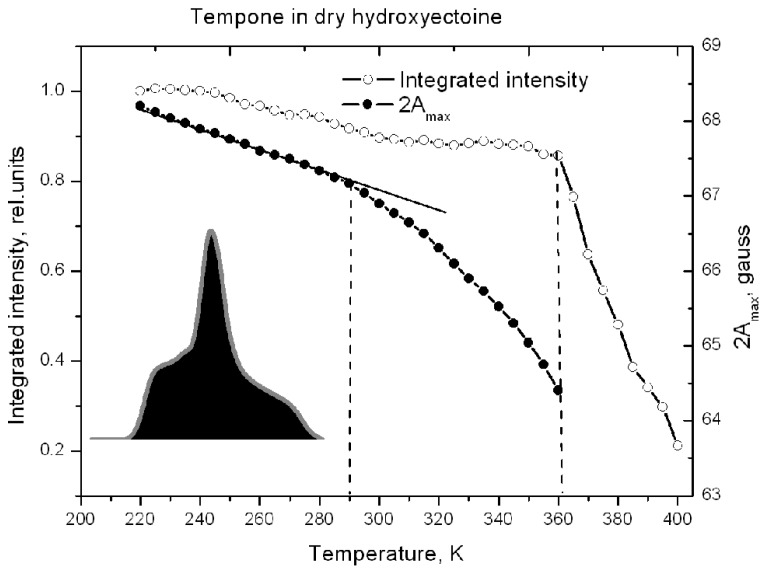
**Temperature dependence of the distance between outermost extremes 2A_max_ (as in Figure 7) and the relative spectral integrated intensity (normalized to the intensity at 220 K) of the ESR spectra of Tempone in dry hydroxyectoine.** Inset – first integral of ESR spectrum (absorption) at 220 K. The integrated intensity is the area (black) under the absorption peak (gray).

**Figure 8** shows that 2A_max_ of Tempone spectra decreases slowly up to 290 K, after which the rate of disordering increases, but the spin probe remains immobile up to 360 K. Obviously, the break in the temperature dependence of 2A_max_ is caused by some structural rearrangements in the solvent matrix (Dzuba et al., 1993, 2005). Above 360 K, 2A_max_ cannot be determined because the spectrum becomes isotropic (**Figure 7**). The ESR spectrum is the first derivative of the absorption spectrum. To estimate the number of paramagnetic centers in a sample, the ESR spectrum has to be double-integrated to obtain the area under the absorption peak (**Figure 8**, inset). The double integral of the ESR spectra is called integrated intensity. The onset of a sharp decrease in integrated intensity coincides with the point of fast isotropic rotation at 360 K. Such an increase of the molecular freedom may result from the breaking of hydrogen bonds between the ketone group of Tempone and the hydroxyl groups of hydroxyectoine.

**FIGURE 9:**
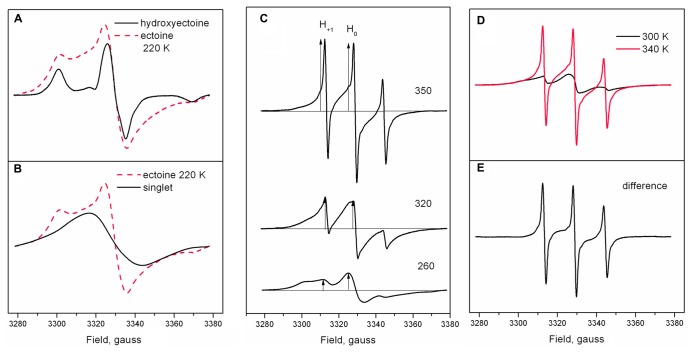
**(A)** Tempone spectra from dry ectoine and hydroxyectoine at 220 K adjusted for spectral intensity and peak position; **(B)** a singlet obtained by subtraction of Tempone spectrum in hydroxyectoine from Tempone spectrum in ectoine as in **(A)**; dashed red line is a Tempone spectrum in ectoine. **(C)** Tempone spectra in dry ectoine at different temperatures. Arrows indicate the position of the central line H_0_ and narrow low-field line H_+__1_. **(D)** Tempone spectra in dry ectoine at 300 and 340 K (red line), adjusted for spectral intensity and line position; **(E)** the difference between spectra in **(D)**.

**Figure 9A** shows the shape of Tempone spectra in dry ectoine and hydroxyectoine at 220 K. The shapes of the spectra are different. The spectrum from hydroxyectoine was subtracted from that in dry ectoine after spectral titration (adjustment of spectral position and amplitudes). The difference spectrum is a singlet (**Figure 9B**). The spectrum subtraction shows that the ESR spectrum of Tempone in ectoine is the superposition of a solid-like triplet (as in hydroxyectoine) and a singlet. The solid-like triplet spectrum (as in **Figures 7** and **9A**) is caused by Tempone molecules, which are spatially separated (i.e., in a glassy state of ectoine). Singlet spectra (**Figure 9B**) are caused by spin exchange between highly concentrated Tempone molecules (Knowles et al., 1976). This results from Tempone molecules, which are excluded from crystalline ectoine and therefore locally concentrated. It is therefore possible to conclude that under the conditions employed (5 days of air-drying at room temperature), solid ectoine samples are a mixture of glassy and crystalline states.

The superposition of singlet and triplet in Tempone spectra in dry ectoine does not allow the correct determination of 2A_max_. However, it is still possible to characterize the temperature-induced dynamic changes in Tempone spectra in dry ectoine at a semi-quantitative level by plotting the ratio of the heights of the positive peaks of the low-field narrow line H_+__1_ and the central line H_0_ (arrows in **Figure 9C**) against temperature (H_+__1_/H_0_ vs. temperature). The central line H_0_ is a superposition of central lines from both solid-like and fluid-like spectra, while the low-field narrow line H_+__1_ represents only the fluid-like spectrum (**Figure 9C**). H_+__1_/H_0_ is an approximate estimation of the proportion of the fluid-like spectral component. Subtraction of the Tempone spectrum of ectoine at 300 K from that at 340 K (**Figure 9D**) shows the narrow-line spectrum, which has a shape typical for fast isotropic motion of the spin probe molecule (**Figure 9E**). **Figures 9C–E** clearly demonstrate the gradual increase of the fluid-like component of the spectra and thus also the increase in the proportion of the freely rotating Tempone molecules in the sample.

**FIGURE 10:**
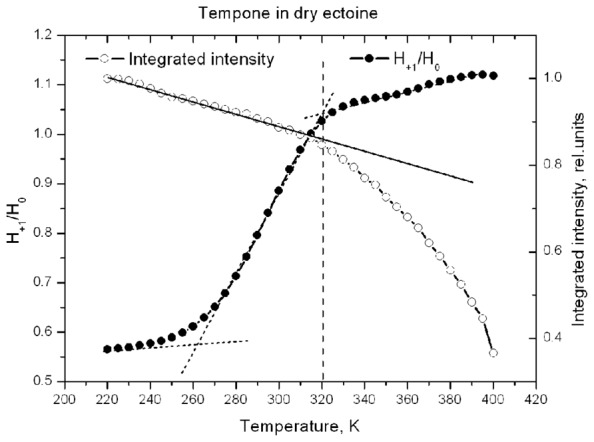
**Temperature dependence of H_+1_/H_0_ and normalized integrated intensity of Tempone spectra in dry ectoine**.

**Figure 10** shows the temperature-induced changes in H_+__1_/H_0_ of Tempone spectra in dry ectoine. In contrast to hydroxyectoine (**Figure 8**), the fluid-like component of Tempone spectra from ectoine appears already around 240–250 K as the increase of H_+__1_/H_0_, but the main changes occur between 270 and 320 K. At T > 320 K the spectral shape is presented mainly by a narrow line isotropic spectrum as in **Figure 9C** top. This point of change (320 K) again coincides with the onset of a decrease in integrated intensity of ESR spectra.

Comparison of data in **Figures 8** and **10** suggests that H-bonds between Tempone (>C=O) and hydroxyectoine exist up to 360 K (87°C) and are completely broken above this temperature allowing free rotation of spin probe molecules. In ectoine the break in hydrogen bonds between Tempone and solute molecules begins below room temperature and is completed at 320 K (47°C). These data show that hydroxyectoine glasses are more thermostable than ectoine glasses.

### FTIR STUDY OF SOLID MATRIX OF ECTOINE AND HYDROXYECTOINE

Spin probe ESR shows only interactions between guest molecule and solute molecules. Temperature-controlled infrared spectroscopy provides information about H-bonding intra- and intermolecular interactions between solute molecules. The FTIR study of dry ectoine and hydroxyectoine was performed parallel to the spin probe study. Visually, partial macro-crystallization of dry ectoine was observed. For IR spectra recording an amorphous area, 500 μm × 500 μm in size, was selected under attached Perkin-Elmer IR- microscope. This partial crystallization of ectoine had already been concluded from the shape of ESR spectra of Tempone in dry ectoine (**Figure 9**). In the case of hydroxyectoine, no visual crystallization of the dry solid was observed under the microscope.

**FIGURE 11:**
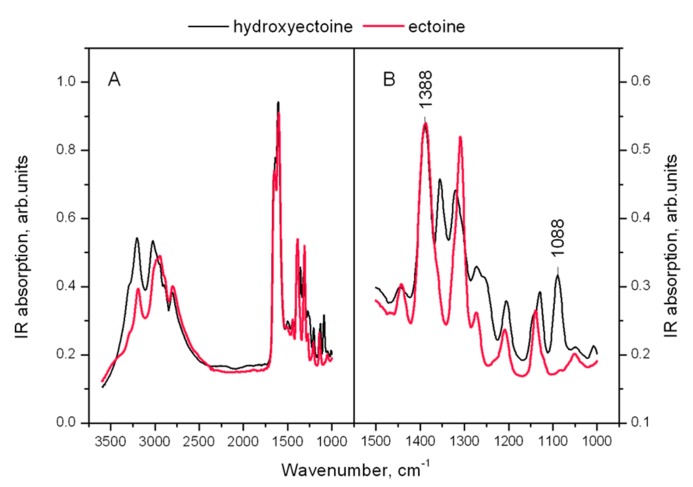
**(A)** FTIR spectra of dry amorphous ectoine (red line) and hydroxyectoine at room temperature. The intensity of the spectra are normalized to the height of the peak at 1600 cm^-1^; **(B)** enlarged finger print region of FTIR spectrum.

**Figure 11A** shows the IR spectra of dry amorphous ectoine and hydroxyectoine. Both spectra contain broad overlapping bands in the hydrogen stretching region N-H, C-H, and N-H (>2500 cm^-1^) and a set of narrow lines in the finger print region (**Figure 11B** in details). The OH stretching vibration band around 3300 cm^-1^ as a function of temperature was previously used to determine Tg in dry sugars and their mixtures (Wolkers et al., 1998, 2004; Kets et al., 2004; Imamura et al., 2006). The overlapping of different broad peaks in the hydrogen stretching region 2500–3500 cm^-1^ in ectoine and hydroxyectoine spectra (**Figure 11A**) does not allow the use of OH stretching band at around 3300 cm^-1^ for characterization of glasses formed by these compounds. The fingerprint region of the FTIR spectra contains several peaks, which are shown in detail in **Figure 11B**. The identification of peaks in the fingerprint region is difficult and needs additional information (Coates, 2000). The fingerprint area of FTIR spectra from dry hydroxyectoine has some additional peaks which are not present in ectoine, and some similar peaks (**Figure 11B**). The differences are caused by the presence of additional OH group in hydroxyectoine, which is attached to the heterocyclic ring.

**FIGURE 12:**
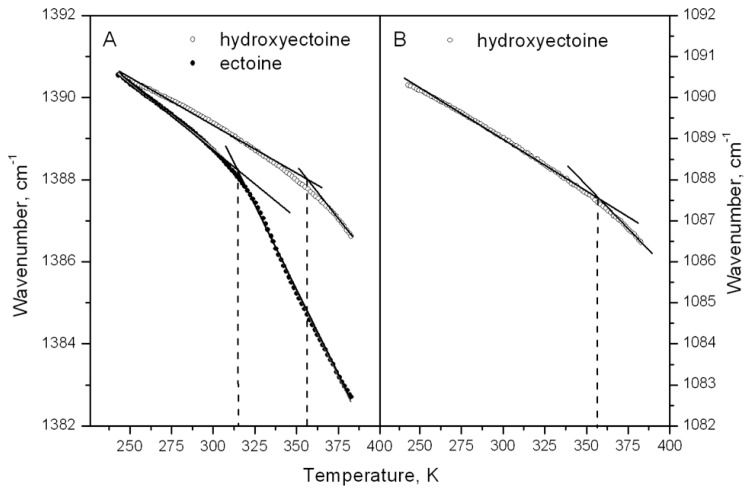
**Temperature dependence of the frequency vibration at 1382–1392 cm^–1^ in dry ectoine and hydroxyectoine (A) and at 1082–1092 cm^–1^ in dry hydroxyectoine (B)**.

The information obtained from ESR experiments (**Figures 8** and **10**) can be used for assignment of some bands in the fingerprint region of the spectra in **Figure 11B**. Our spin probe study showed that the H-bonds between >C=O of Tempone and OH- group of dry hydroxyectoine are broken at approximately 360 K. The inspection of the wavenumber-temperature dependences of all major peaks in the fingerprint area of hydroxyectoine showed that only the bands around 1088 and 1388 cm^-1^ (**Figure 11B**) have a break at approximately 360 K (**Figures 12A,B**). The peak around 1088 cm^-1^ does not exist in the ectoine spectrum, and can thus be attributed to OH group attached to heterocyclic ring in hydroxyectoine. On the other hand, the FTIR spectrum of dry ectoine has the same band around 1388 cm^-1^ as hydroxyectoine (**Figure 11B**) and can be assigned to NH groups in both ectoine and hydroxyectoine. However, the break in the wavenumber-temperature dependence for this band in ectoine occurs not at 360 K, as in hydroxyectoine, but at approximately 320 K (**Figure 12A**). This coincides with the temperature at which all Tempone molecules start free rotation (**Figures 9** and **10**). The fact that IR inflections of hydroxyectoine are less distinct, is explained by experimental limitations (i.e., upper temperature limit of 100°C und, therefore, fewer data points beyond Tg) and higher strength of OH hydrogen bonds resulting in a less pronounced slope of the wavenumber vs. temperature plot.

## DISCUSSION

### PROTEIN STABILIZATION EFFECTS OF COMPATIBLE SOLUTES IN SOLUTION

Compatible solutes (organic osmolytes) of halophilic bacteria have long been known as versatile stress-protecting compounds, in particular for the stabilization of proteins and whole cells. The molecular nature of their stabilizing function has been explained by preferential interaction with water and subsequent exclusion from a proteins hydration shell (Arakawa and Timasheff, 1985). This original concept has been expanded by others, who revealed that the unfavorable interaction of the peptide backbone is the main driving force for this stabilization phenomenon, named the “osmophobic effect” (Bolen and Baskakov, 2001). In order to elucidate the molecular features which make a solute a stabilizing compound, a quantitative solvation model was proposed (Street et al., 2006), in which backbone/solute interaction energy is a function of the interactants’ polarity and surface area. On the basis of this model, we are now able to quantify the chemical features, which make a solute “compatible” and rank them according to their protein stabilization in solution. The superiority of hydroxyectoine over ectoine in desiccation protection of cells and biomolecules has, however, so far not been explained.

### ESR STUDIES PROVE SUPERIOR GLASS PROPERTIES OF HYDROXYECTOINE

The presented ESR data show that dry hydroxyectoine has considerable advantages as a matrix for guest molecules over ectoine. Hydroxyectoine forms stable glasses, while ectoine is more prone to crystallization. The difference is caused by one additional OH group in hydroxyectoine. This group reduces the degree of molecule symmetry and thus probably prevents the molecules from tight packing (Wang et al., 2009). This creates the space for optimized orientation of molecules for H-bonding, the formation of which needs not only the presence of donor and acceptor, but also specific geometry (Steiner, 2002). The more bulky shape of hydroxyectoine in dry matrix provides better conditions for H-bonding with other molecules than the more symmetric ectoine, which has a tendency to aggregate and crystallize. As a result, the additional hydroxyl provides more possibilities for H-bonds by increasing the number of donors, the strength of intermolecular interactions and the energy barrier for the reorganization of the molecules. It has already been shown that the presence of hydroxyl groups increases thermal stability of glasses (Omayu et al., 2008; Zhou et al., 2013).

The large temperature difference between molecular fluidizing in ectoine (320 K) and hydroxyectoine (360 K) of 40 degrees, as shown by ESR and FTIR studies (**Figures 8**, **10**, and **12**), results from different types of intermolecular H-bonds that cause molecular immobilization. In ESR experiments, the motional behavior of Tempone in hydroxyectoine is determined by the strongest H-bonds between CO of Tempone and OH of the solute. In ectoine, motional behavior of Tempone is determined by weaker H-bonds with NH groups of the solute.

Stronger H-bonds provide not only better structural stability but also chemical stability of Tempone molecules in dry hydroxyectoine. Chemical stability of dry material is associated with molecular immobilization, which considerably reduces the probability of diffusion-controlled reactions. The probability of chemical reactions increases above glass transition (Craig et al., 2001). The spin probe approach provides an opportunity to obtain information about molecular immobilization and redox activity from the same ESR spectra. Spin probe molecules can participate in different redox-reactions resulting in non-paramagnetic species. The most probable are reduction to hydroxylamine and irreversible reaction with other radicals to stable non-radical products (Belkin et al., 1987). In the case of redox conversion of spin probe molecules, the number of paramagnetic species decreases resulting in the decrease of the integral intensity of the spectra (**Figures 8** and **10**).

The integrated intensity of the ESR spectra of Tempone in dry hydroxyectoine does not significantly change up to the point of breaking H-bonds between 360 and 365 K. Above this temperature, the integrated intensity of the spectra sharply decreases (**Figure 8**). This coincides with the transformation of the spectral shape of Tempone to fluid-like type (**Figure 7**). The break in the temperature dependence of the integrated intensity of Tempone spectra in dry ectoine was observed at 320 K (**Figure 10**). As in the case of hydroxyectoine, the redox conversion of Tempone in dry ectoine sharply increases when spin probe molecules obtain a motional freedom similar to that in a fluid phase (**Figure 9E**). Such motional freedom was obviously caused by the break of H-bonds >C=O...-HN (>CO belongs to Tempone, NH belongs to ectoine). Clearly, redox conversion of spin probe molecules becomes possible when they obtain motional freedom. The fluid-type spectrum of Tempone indicates the possibility of translational diffusion and increased probability of chemical reactions. Therefore, the presence of hydroxyectoine in the dry cytoplasm of anhydrobiotic organisms would improve the structural and chemical stability of glasses as a result of increased number and strength of H-bonds between molecules.

### FTIR STUDIES CONFIRM GLASS TRANSITION TEMPERATURES OF ECTOINE AND HYDROXYECTOINE

The broad region of OH stretching vibrations around 3380 cm^-1^ in FTIR spectra is commonly used to determine Tg in dry sugars and their mixtures (Wolkers et al., 1998, 2004; Kets et al., 2004; Imamura et al., 2006). Ectoine does not contain OH groups and therefore does not contain a distinct band in this region (**Figure 11A**). Hydroxyectoine contains one OH group attached to the heterocyclic ring, and the FTIR spectrum is expected to display the band around 3380 cm^-1^ from OH stretching vibrations as for dry sugars. However, only a weak shoulder is present in this region in the FTIR spectrum of hydroxyectoine (**Figure 11A**). Obviously, the broad bands from NH stretching and CH stretching vibrations (2800–3200 cm^-1^) mask the OH band at 3380 cm^-1^. Close inspection of bands within the fingerprint region helped to find other bands, which can be used to characterize glass transition in dry (hydroxy)ectoine. The decrease of the wavenumber with temperature for both bands at 1388 and 1088 cm^-1^ (**Figures 12A,B**) is characteristic for bending deformations of the functional groups, which are involved in H-bonding (Vanderkooi et al., 2005). All data together allow the assignment of the band around 1388 cm^-1^ to bending deformations of NH groups of the ring, and the band around 1088 cm^-1^ to bending deformations of OH attached to the heterocyclic ring. The same position of the break of the temperature dependence for OH and NH bending vibrations in hydroxyectoine (360 K, **Figures 12A,B**) indicates that in this dry matrix NH groups form intermolecular H-bonds with OH groups. In ectoine, OH groups are absent and such H-bonds are not possible. Weaker intermolecular H-bonds between NH and CO of COO^-^ group and, even weaker still, H-bonds between NH groups determine the lower position of the break in the temperature dependence of the wavenumber of NH bending vibration in dry ectoine (**Figure 12A**).

In conclusion, it can be said that FTIR data corroborated the ESR observations, which demonstrate a remarkable difference in Tg of approximately 40°C between ectoine and hydroxyectoine. The high Tg of the latter (87°C) places hydroxyectoine somewhere between sucrose (65°C) and trehalose (117°C).

### RESPONSE OF *H. elongata* TO SALT AND TEMPERATURE STRESS

Earlier studies have shown increasing hydroxyectoine levels in response to salinity and temperature (Wohlfarth et al., 1990; Severin et al., 1992) in the *Halomonadaceae*. This upregulation of hydroxyectoine synthesis has subsequently been investigated in greater depth with the related *C. salexigens*. Under comparable growth conditions (1.5 M NaCl = 8.7% and 2.5 M NaCl = 14.5%, at a temperature of 45°C), relative proportions of hydroxyectoine of approximately 60 and 70% respectively have been reported (Garcia-Estepa et al., 2006; Rodríguez-Moya et al., 2013). By genetic manipulation (transcriptional fusion, gene duplication, and overexpression on vector), this proportion was increased even further to 77% (8.7% NaCl, 37°C), albeit at the expense of a reduced growth rate (Rodríguez-Moya et al., 2013). As demonstrated by **Figure 2**, *H. elongata* grown at 15% NaCl similarly increases its relative proportion of hydroxyectoine in response to both salinity and temperature, with a maximum of approximately 70% at the highest tolerated growth temperature of 45°C. However, we report here for the first time that a sudden temperature upshift to 50°C (beyond the organism’s temperature maximum) also leads to a rapid increase in the proportion of hydroxyectoine (up to 75%). Surprisingly, such a temperature shift, when applied in the exponential phase, did not markedly impair growth and yield (**Figure 3**).

### DESICCATION PROTECTION OF WHOLE CELLS

Given the superior glass properties of hydroxyectoine, it is not surprising that (water-stressed) halophilic ectoine-type organisms have adopted a rather economical strategy to protect themselves from desiccation damage. Instead of synthesizing distinct glass-forming compounds such as sucrose and/or trehalose, they simply convert, by a single enzymatic step, one compatible solute into another, hydroxyectoine, with a much higher Tg. The consequences of such a conversion from 17 to 75% hydroxyectoine (experimentally enforced by salt and temperature shock) is clearly demonstrated by a six-times increase in desiccation survivors under drastic drying conditions (3 h at 45°C and 10 mbar; **Figure 4**). This remarkable improvement was probably accomplished by the high cytoplasmic concentrations of intracellular glass-forming hydroxyectoine. Desiccation tolerance of the related *C. salexigens* has been investigated with cells grown under similar conditions (14.5% NaCl and 45°C) and applying the drying protocol by Manzanera et al. (2002), which entails vacuum drying at 30°C and 313 mbar for 30 h (Reina-Bueno et al., 2012). These conditions are less drastic than those applied here, except for length of time. The authors observed a surprisingly low desiccation tolerance of approximately 5% survivors, which was reduced even further by at least one order of magnitude in knock-out mutants unable to synthesize either hydroxyectoine or trehalose. From this they concluded that both compounds probably play a role in desiccation tolerance, although other factors must be considered. As the relative hydroxyectoine content in *C. salexigens* is similar, the much better performance of *H. elongata* is either explained as a consequence of more favorable drying conditions or of other factors triggered by temperature upshock (see below).

As we did not want to exceed the maximum growth temperature during the drying process, the temperature applied experimentally happened to be close to the Tg of ectoine (47°C), but much below that of hydroxyectoine (87°C). One would therefore assume that a slightly increased drying temperature (e.g., 50°C) would enhance the difference between ectoine and hydroxyectoine as desiccation protectants.

The experimental set-up presented here enforced the cytoplasmic conversion of ectoine into hydroxyectoine, and purposely avoided the use of external excipient as an additional protective element, although one would expect a positive influence from additional external protection. Others have improved the desiccation tolerance of whole cells (both *E. coli* and *P. putida*) by external application of 1 M trehalose or hydroxyectoine, both in combination with 1.5% polyvinyl pyrollidone (PVP) as a thickening agent (Manzanera et al., 2002, 2004a). For *E. coli*, they obtained survival rates of approximately 60% after vacuum drying with very little difference between trehalose and hydroxyectoine. In the case of *P. putida*, hydroxyectoine (approximately 40% survival) performed even better than trehalose (<20% survival). Hence the positive effect of hydroxyectoine as an external drying excipient (comparable to that of trehalose) appears to be met by a similarly striking effect when accumulated intracellularly. It remains to be shown whether the external addition of glass-formers (i.e., a combination of both strategies) would increase the survival rates even more (de Castro et al., 2000).

### OTHER POTENTIAL FACTORS INVOLVED IN DESICCATION TOLERANCE

Inorganic ions, and in particular polyphosphates, are suspected of also playing a role in stress adaptation, either alone or in combination with other solutes (Seufferheld et al., 2008). All attempts using HPLC and ^31^P-NMR techniques to detect any major changes in inorganic cations (Mg, Ca, Na, K) and anions (Cl, NO_3_, SO_4_, PO_4_), including polyphosphates, were however, unsuccessful. It therefore appears that the conversion of ectoine into hydroxyectoine is the major observable change within low-molecular mass compounds when *H. elongata* cells prepare for a pending heat stress/desiccation event. The notable exception is potassium glutamate, the only other organic compound present in large amounts (ratio of approximately 1:4 glutamate/hydroxyectoine). Glutamate accumulation is one of the first physiological responses to salinity-induced dehydration in many microorganisms. Whether its H-donor and H-acceptor groups also contribute to the formation of a stabilizing H-bond network in the glassy state still needs to be clarified. These findings indicate that in particular hydroxyectoine is the crucial low-molecular mass compound for desiccation survival of *H. elongata*. It seems that the presence of at least one such glass-forming compound is essential to enable anhydrobiotic stabilization of biological systems (Tunnacliffe et al., 2001).

We cannot of course exclude that, besides hydroxylation of ectoine, other factors triggered by the combination of high salt and temperature upshock, such as heat-stress proteins, may play a role in increased desiccation protection. We were, however, unable to detect any temperature-induced proteins at the expected molecular mass. Similarly, very small IDPs (2–30 kDa) may also play a role. Among these, late embryogenesis abundant (LEA) proteins, essential for glass formation in dormant seeds, are of particular interest (Tunnacliffe and Wise, 2007). These proteins are highly charged (due to the abundance of glutamate and lysine residues), highly hydrated and form random coils in solution. During desiccation, the formation of secondary structures releases water. Therefore, IDPs probably serve as water-retaining molecules and as scavengers for inorganic ions (ion sequestration). As such they slow down removal of water and prevent adverse effects of increased ionic strength (Tompa et al., 2006). Garay-Arroyo et al. (2000) coined the term “hydrophilins,” an expanded definition of IDPs which comprises LEA proteins and dehydrins. Such proteins are characterized by a hydrophilicity index >1 and a glycine content >6% as a distinguishing feature. In *E. coli*, five proteins conform to this definition (four of which respond to osmotic stress; Garay-Arroyo et al., 2000). One of the *E. coli* hydrophilins (YCIG) has been checked for its stabilization properties with LDH during vacuum drying, and performed well (comparable to 100 mM trehalose) at a molar ratio of 1:1 (hydrophilin:enzyme) up to 98% water loss (Reyes et al., 2005). A bioinformatic study on the *H. elongata* genome revealed only four uncharacterized small proteins which conform to the above criteria of hydrophilins (unpublished); however, a possible involvement in desiccation response has not yet been shown. We therefore have at present no proof that small water-binding/ ion sequestering proteins, as discussed for desiccation tolerant plants, invertebrates, cyanobacteria, fungi, and other anhydrobiotic organisms (Hand et al., 2011), may also participate in the desiccation protection of the moderately halophilic, extremely halotolerant *H. elongata*.

### AIR-DRYING OF MODEL ENZYME LACTATE DEHYDROGENASE AT HIGH TEMPERATURE

The parameters of the drying process applied to model enzyme LDH were chosen so as to display the consequences of the vastly different Tg of the two ectoines. The drying temperature of 60°C lies above the Tg of ectoine (47°C) but below that of hydroxyectoine (87°C). The concentration of the enzyme (50 μg/mL) was chosen to minimize self-stabilizing effects (Lippert and Galinski, 1992) and excludes the interference of stabilizers from the commercial preparations. Under these conditions, hydroxyectoine performed very well (70% residual activity, as compared to approximately 60% for sucrose and approximately 80% for trehalose) after 2 h drying (**Figure 5**). These values compare with those previously obtained after freeze-drying in 1 M solution, all approximately 70% (Lippert and Galinski, 1992), and those of a comparative experiment with hydrophilins and trehalose when vacuum-dried down to 2% residual water, approximately 80%, as described by Reyes et al. (2005). Delete the part “(albeit at an even lower enzyme concentration)” . The most striking difference here, however, lies in the fact that ectoine had no stabilizing effect whatsoever. This is very likely due to the high drying temperature, well above the Tg of ectoine. The fact that prolonged drying (4 and 6 h) reduced residual activity, and in the case of hydroxyectoine, more severely than in the presence of sucrose and trehalose, may be explained by differences in their water-retaining capacity. As has been shown in vacuum-drying experiments of LDH, the final water content has a great influence on residual activity, which rapidly decreases beyond 97% water loss (Reyes et al., 2005). The water-retaining capacity of hydroxyectoine has so far not been investigated, whereas the remarkable anhydrobiotic properties of trehalose have been studied in depth and accredited to its unusual polymorphism (several transitions between polymorphic crystalline and amorphous states). In particular, the dihydrate crystallites allow diffusion of water in and out of channels (Jones et al., 2006). The crystalline-glassy nanocomposite matrix of trehalose traps residual water molecules and makes it such a good material for the preservation of protein conformation (Jain and Roy, 2009; Sussich et al., 2010).

Without doubt the kinetics of water removal have a strong influence on the formation of the glassy state, and in particular the amount of retained water (Willart et al., 2002; Cesàro, 2006). This becomes also apparent when the results of the air-drying of a droplet of solute are closely investigated. Not only does this depend on the hydrophobicity of the surface, which alters microflow patterns and influences crack formation upon continued drying (Adams et al., 2008), but also on droplet size and the speed of water removal at the air-liquid interface. The evaporation of solvent creates steep solute concentrations and temperature gradients, which in turn yield aggregation of solutes, skin formation, crystallization, and most importantly, a spatial heterogeneity in the desiccated droplet (Ragoonanan and Aksan, 2008). In addition, shearing forces (resulting from crystallization effects) and mechanical stress (from cracking) may also damage biological structures in the dry state (Aksan and Toner, 2004; Jones et al., 2006). All these physical, rheological and chemical factors, when combined, tend to create an almost unpredictable outcome. As the glass-forming properties of hydroxyectoine, although suspected for a long time, have only now been proven, we still need to resolve to what extent speed of drying and residual water content influence its stabilizing effect on biomolecules and whole cells. Nevertheless, we are now able to present a novel glass-forming desiccation protectant, hydroxyectoine, which may even be able to challenge the best-known sugar-type stabilizers.

## Conflict of Interest Statement

The authors declare that the research was conducted in the absence of any commercial or financial relationships that could be construed as a potential conflict of interest.
